# Association between Sleep and Language Development in Children with Congenital Zika Syndrome

**DOI:** 10.3390/v16071003

**Published:** 2024-06-22

**Authors:** Nathani C. da Silva, Celia M. Giacheti, Maria C. H. do Couto, Stefany S. de Jesus, Erlane M. Ribeiro, Islane M. C. Verçosa, Luciana Pinato

**Affiliations:** 1 Department of Speech, Language and Hearing Sciences, São Paulo State University (UNESP), Marília 17525-900, Brazil; nathani.cristina@unesp.br (N.C.d.S.); c.giacheti@unesp.br (C.M.G.); clara.couto@unesp.br (M.C.H.d.C.); stefany.santos@unesp.br (S.S.d.J.); 2 Albert Sabin Children’s Hospital, Fortaleza 60410-790, Brazil; erlaneribeiro@yahoo.com.br; 3 Center for Perfecting Sight See Hope Reviver (CAVIVER), Fortaleza 60110-370, Brazil; islaneverc@gmail.com

**Keywords:** neurodevelopment, sleep, language, syndrome, zika virus infection

## Abstract

AIM: Congenital Zika Virus Syndrome (CZS) presents notable hurdles to neurodevelopment, with language development emerging as a crucial aspect. This study investigates sleep patterns and language skills in children with CZS, aiming to explore the potential synchronization of sleep development with their neurodevelopment. METHOD: We studied cross-sectionally 135 children with CZS aged 0 to 48 months, investigating sleep using the BISQ Questionnaire. Language development was assessed using the Early Language Milestone Scale, while motor development and cognitive and social ability were assessed using the Bayley Scales of Infant and Young Child Development 3rd edition. We also studied longitudinally a cohort of 16 children (initially aged 0 to 12 months) whom we followed for four years, assessing at one-year intervals. RESULTS: Sleep disturbances and language deficits were highly frequent in this population. In the 0–12 months group, a late bedtime and frequent nighttime awakenings were associated with poorer auditory expressive skills. At 13–24 months, nighttime awakenings were associated with poorer auditory expressive skills, while among 25–36-month-olds decreased auditory receptive skills were associated with longer sleep onset latency and reduced nighttime sleep duration. CONCLUSION: The brain alterations caused by Zika virus infection affect both sleep disturbances and delays in language development. It is possible that sleep disturbance may be a mediating factor in the pathway between CZS and delayed language development, as the three analyzed language skills showed a correlation with sleep parameters.

## 1. Introduction

In 2015, the Zika virus (ZIKV) emerged in the Americas and spread to 87 countries and territories with autochthonous transmission, covering four of the six regions of the World Health Organization (WHO) [1]. When ZIKV infections occurred during the gestational period, they were, in many cases, transmitted from the mother to the fetus, resulting in serious malformations, especially when they occurred during the first trimester of pregnancy [2].

These children, exposed to ZIKV during the intrauterine period, were born with a set of structural anomalies and functional impairments primarily due to damage to the central nervous system during the formative period, which has been termed Congenital Zika Syndrome (CZS), with the most common clinical manifestation being microcephaly [3]. As this is the first group of children to manifest this condition, their follow-up becomes crucial for understanding all the potential neurological impairments of the syndrome that will manifest throughout their lives.

Among the phenotypic characteristics found in the first year of life are irritability, convulsions, clonus, crying, epilepsy, dysphagia, neurodevelopmental cognitive and language disorders, motor disorders with congenital contractures, hypertonia, extrapyramidal disturbances, and sleep disturbances [3,4].

The first study to investigate sleep complaints in this population demonstrated that 12-month-old babies had a significantly higher percentage of sleep problems, with total sleep duration and nighttime sleep time being lower compared to children with typical development [4]. Other studies have confirmed a high frequency of sleep disorders in this population [5,6].

Regarding potential neurological alterations related to sleep disturbances, studies are still incipient and should explore this issue in the future. The neuroimaging features of microcephaly secondary to Zika virus (ZIKV) infection are extensive and varied, reflecting the severe impact of the virus on brain development [7,8].

Key findings from imaging studies, such as MRI and CT scans, include calcifications at the junction of gray–white matter and subcortical white matter, associated cortical abnormalities, and a general diminution of white matter. Other frequent abnormalities include ventriculomegaly, corpus callosum hypoplasia, subependymal cysts, hypoplasia of the cerebellum and brainstem, and enlargement of the cerebellomedullary cistern [7,8].

Extensive calcifications are a hallmark of congenital Zika syndrome, predominantly observed at the gray–white matter junction, but also in the thalamus and basal ganglia. Abnormalities in cortical development are frequent, occurring in 94–100% of cases, with common presentations including agyria–pachygyria. These abnormalities are usually diffuse, predominantly affecting the frontal, insular, and parietal lobes, and are often accompanied by wide Sylvian and interhemispheric fissures [7,8].

Despite these detailed imaging findings, few studies have explored the correlations between neuroimaging abnormalities and the clinical symptoms in affected children. For instance, Aragão et al. showed that arthrogryposis correlates with greater severity of brain damage, indicated by a higher number of cerebral calcifications, a greater likelihood of infratentorial calcifications, and brainstem and cerebellar hypoplasia [9].

Furthermore, Nunes et al. highlighted significant EEG abnormalities in children with ZIKV-related microcephaly. The EEG background was abnormal in 72.1% of cases when awake and in 62.8% during sleep, with significant differences in EEG patterns between different neuroimaging groups. Children with milder neuroimaging findings were more likely to have normal sleep EEG patterns [10].

Sleep–wake alterations should receive early attention, since the establishment of a sleep pattern in the first months of life is of fundamental importance for the development of newborns. During sleep, the body releases hormones that play a crucial role in children’s development and weight gain [11]. Adequate sleep contributes to healthy physical growth, development of muscle mass, and strengthening of the body’s defenses against diseases, which is particularly important for growing children [11,12].

Sleep can also influence neurodevelopment, considering that during the early years of life, a baby’s brain is in a stage of rapid growth and development, dependent on genetic and social and physical environmental signals, and sleep plays a role in maintaining homeostasis at each moment, allowing for the maturation of new functions over time [12,13].

With a crucial role in memory consolidation, the organization of neural connections, and the regulation of hormones essential for healthy development, regular and adequate sleep allows a newborn’s brain to process and store information crucial for learning [14]. Furthermore, sleep plays a vital role in the restoration and repair of the nervous system, promoting brain health and recovery from any potential injuries or metabolic stress.

The consequences of sleep disorders and other circadian rhythm disturbances can pose obstacles to the neurological development of the child, resulting in cognitive problems and learning difficulties, and can also influence emotional and behavioral development [15,16]. Therefore, establishing a healthy sleep pattern in the first months of life is crucial for providing a solid foundation for proper neurodevelopment in newborns.

The severity of sleep disorders also reflects on the health and quality of life of the parents, with negative repercussions for the parents’ ability to properly apply treatment techniques and learned strategies [4].

The follow-up studies conducted so far have shown that several phenotypic characteristics demonstrated at birth have remained persistent over time in these children, including severe and long-lasting impairments in language development, with no progression of linguistic skills between 12 and 36 months of age [17]. As for the development of sleep patterns, this knowledge remains a gap to be filled in our understanding of this syndrome.

In order to investigate whether sleep problems are really among the striking and persistent characteristics of the phenotype of this syndrome, the present study aims to carry out a longitudinal analysis of sleep and investigate its possible association with language development.

## 2. Materials and Methods

We studied cross-sectionally 135 children diagnosed with CZS, of both genders, aged 0 to 48 months, subdivided into 4 age ranges (0–12; 13–24; 25–36; 37–48 months). All the participating children are part of a group served by a non-governmental organization in the city of Fortaleza, Brazil. These children reside in Fortaleza or other cities in the state of Ceará, Brazil, and are seen semiannually at this facility by a multidisciplinary team. All children diagnosed with CZS who attended the multi-disciplinary service sessions were evaluated and agreed to participate in this study. Considering that not all children were able to attend every multidisciplinary service session from birth to 48 months, the number of participants (N) per age group varies. 

From those who participated in all the evaluations across all age groups, for the longitudinal analysis of sleep, a cohort of 16 children (initially aged 0 to 12 months) diagnosed with CZS was followed for 4 years and assessed at 12-month intervals. The inclusion criteria for this group were that the children from the total group of 135 had to have been evaluated with the study instruments at all the proposed age intervals.

The recommendations of the National Health Council Resolution (CNS 466/2012) on Guidelines and Regulatory Standards for Research Involving Human Beings were followed, and the study was approved by the Research Ethics Committee of the Albert Sabin Children’s Hospital, Fortaleza, Brazil, under protocol 1.743.023; written informed consent for participation and publication has been obtained for all carers of the individuals. All children participating in the project underwent evaluation by a multidisciplinary team that included ophthalmological, psychological, and complementary examinations. The diagnosis was confirmed by a neurologist and a geneticist.

Sleep was assessed using the Brief Infant Sleep Questionnaire (BISQ). The BISQ assesses the presence of sleep disturbances in children based on their sleep patterns in the last week. It includes questions addressing the following behavioral aspects of sleep: duration of nighttime and daytime sleep, number of nighttime awakenings, time awake during the night, time to fall asleep, method of falling asleep, sleep location, and preferred sleeping position. The criteria established by BISQ to define whether a child has a sleep disorder are as follows: waking up more than three times during the night, staying awake for more than one hour, and a total sleep time of less than nine hours. According to the BISQ, the presence of at least one of these criteria indicates a sleep disorder [18]. 

One of the data points extracted from this questionnaire is the total sleep time. Using this data, we calculated the percentage of children in each age group who sleep less than the recommended amount for their age, according to the guidelines set by the American Academy of Sleep Medicine (AASM) [19].

Language development was assessed using the Early Language Milestone Scale (ELM Scale), which is used to screen language development in children aged 0 to 48 months. It is considered a concise language assessment tool in which major language milestones are grouped into three areas: receptive auditory function, expressive auditory function, and visual function. Language development is considered typical when the ceiling value in all three functions (expressive auditory, receptive auditory, and visual skills) corresponds to the child’s chronological age. This analysis generates a score for each skill, and in this case, the lower the score, the further the child is from the expected performance level for their age [20].

For the investigation of child development skills, the Bayley Scales of Infant and Toddler Development (Bayley-III) which assesses the progressive functional development of children aged 0 to 42 months was used [21]. The Bayley-III is an inventory of behavior observation, designed to be completed by the examiner and the caregiver, which provides information based on observations made during the test. It covers domains of development divided into 5 scales, i.e., Motor, Cognitive, Language, Socioemotional, and Adaptive Behavior. 

The comparison of sleep parameters between different age ranges was conducted using the Kruskal–Wallis test. Correlation analyses between sleep and language performance, sleep and development skills, and language performance and development skills were carried out using the Spearman correlation test, and the significance level adopted was *p* < 0.05.

## 3. Results

A total of 70% of mothers were infected with ZIKV in the first trimester, 22% in the second, and 8% in the third trimester of pregnancy. Approximately 4.8% of the children were born premature and 95.2% were born full-term. All children presented with microcephaly, with 23% classified as severe. The mean ± standard deviation (SD) of head circumference at birth was 29.7 ± 2.1 cm (range 25 to 32 cm), with a birth weight of 2722 ± 484 g and height of 45.2 ± 3 cm; 82% of the children had seizures (Table 1). Hearing loss detected in audiological exams was an exclusion criterion for the sample. Four children with hearing loss were excluded from this study.

Regarding the qualitative analysis of sleep duration, approximately 80% of the children exhibited total sleep times below the recommended guidelines [18], with no significant variation across age ranges (Table 2).

**Figure 1:**
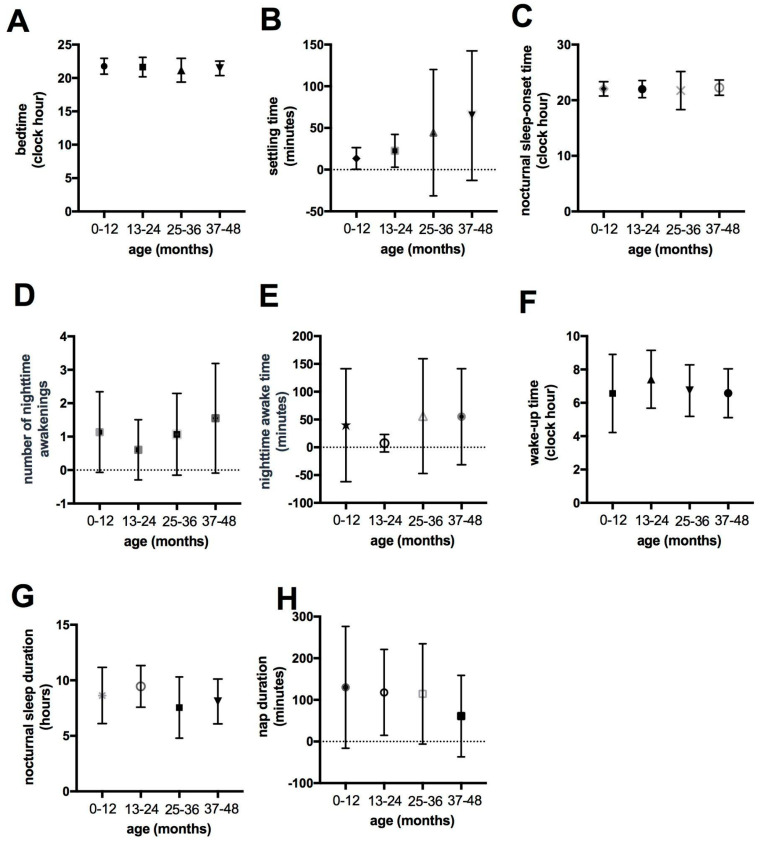
Cross-sectional comparison of sleep parameters in children with Congenital Zika Virus Syndrome across four different age ranges (from 0 to 12 months [N = 22], from 13 to 24 months [N = 33], from 25 to 36 months [N = 60], from 37 to 48 months [N = 20]). (**A**) The time the child goes to bed (bedtime). (**B**) The time it takes for the child to initiate sleep (settling time). (**C**) The nocturnal sleep-onset time. (**D**) The number of nighttime awakenings. (**E**) The night time awake time. (**F**) The wake-up time. (**G**) The nocturnal sleep duration. (**H**) The daytime sleep duration (nap duration).

**Figure 2:**
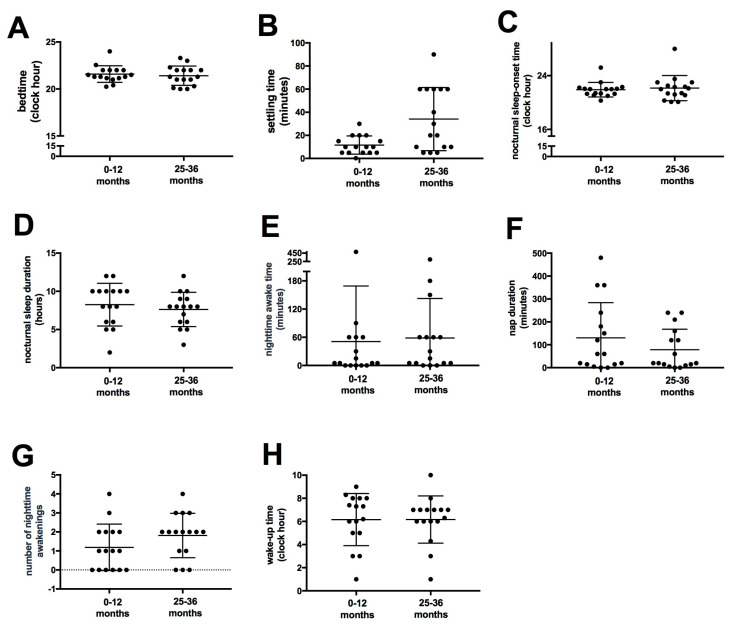
Longitudinal change in sleep parameters in the same children with Congenital Zika Virus Syndrome between successive age ranges (from 0 to 12 months, from 25 to 36 months). (**A**) The time the child goes to bed (bedtime). (**B**) The time it takes for the child to initiate sleep (settling time). (**C**) The nocturnal sleep-onset time. (**D**) The number of nighttime awakenings. (**E**) The night time awake time. (**F**) The wake-up time. (**G**) The nocturnal sleep duration. (**H**) The daytime sleep duration (nap duration). N = 16.

The cross-sectional analysis of sleep parameters did not reveal material differences between children in the four age groups examined (Figure 1). The longitudinal analysis showed that when considering the same group of 16 children across different age groups, there was no disparity in the group medians for any of the parameters analyzed among the different age groups (bedtime set by parents, sleep onset latency, bedtime, nighttime sleep duration, number of awakenings during nighttime sleep, wake-up time in the morning, time awake at night, and daytime sleep hours) (Figure 2).

Describing longitudinal changes (Table 3), we observe the following: 

Nocturnal sleep duration: for 50% of the children, there was a decrease in total nighttime sleep hours from 12 to 36 months; 31.2% maintained the same sleep duration, while 18.7% increased their nighttime sleep.

Sleep latency: a majority (56.2%) experienced an increase in sleep latency from 12 to 36 months, indicating a longer time to fall asleep; 12.5% decreased their sleep latency, and 31.2% remained consistent.

Nighttime awakenings: 43.7% of the children had an increase in the number of nighttime awakenings; half (50%) maintained the same frequency of awakenings, while a small percentage (6.2%) decreased their nighttime awakenings.

Time awake at night: Similarly, 43.7% of the children increased their time awake at night. A quarter (25%) maintained the same duration of time awake, and 31.2% reduced their time awake at night.

Regarding the overall development of these children, none of them showed significant improvement in the assessed parameters.

**Figure 3:**
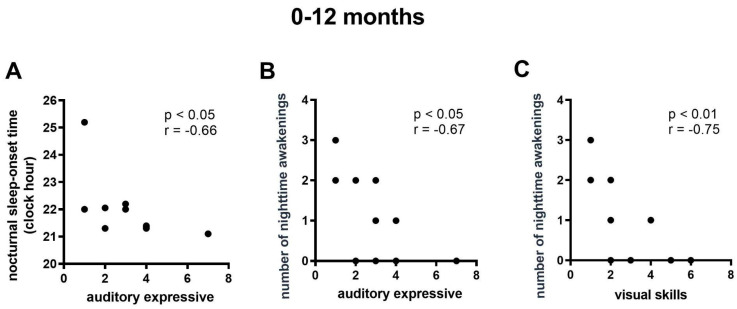
Correlations between the sleep and language parameters among CZS children aged 0 to 12 months. (**A**) Correlation between nocturnal sleep onset time and auditory expressive skills. (**B**) Correlation between number of nighttime awakenings and auditory expressive skills. (**C**) Correlation between number of nighttime awakenings and visual skills. N = 11.

Among children aged 0 to 12 months, the correlation analyses revealed a negative relationship between expressive auditory ability and bedtime as well as nighttime awakenings (Figure 3A,B), and a negative relationship between visual ability and nighttime awakenings (Figure 3C).

**Figure 4:**
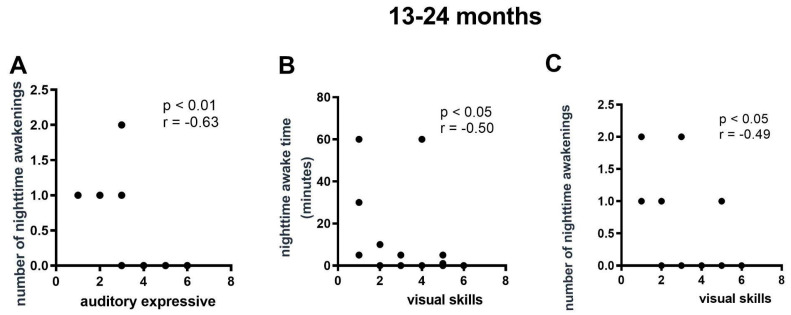
Correlations between the sleep and language parameters in CZS infants with ages ranging from 13 to 24 months. (**A**) Correlation between number of nighttime awakenings and auditory expressive skills. (**B**) Correlation between nighttime awake time and visual skills. (**C**) Correlation between number of nighttime awakenings and visual skills. N = 19.

Among children aged 13 to 24 months, the correlation analysis revealed a negative relationship between expressive auditory ability and nighttime awakenings (Figure 4A), and a negative relationship between visual ability and nighttime wake time and nighttime awakenings (Figure 4B,C).

**Figure 5:**
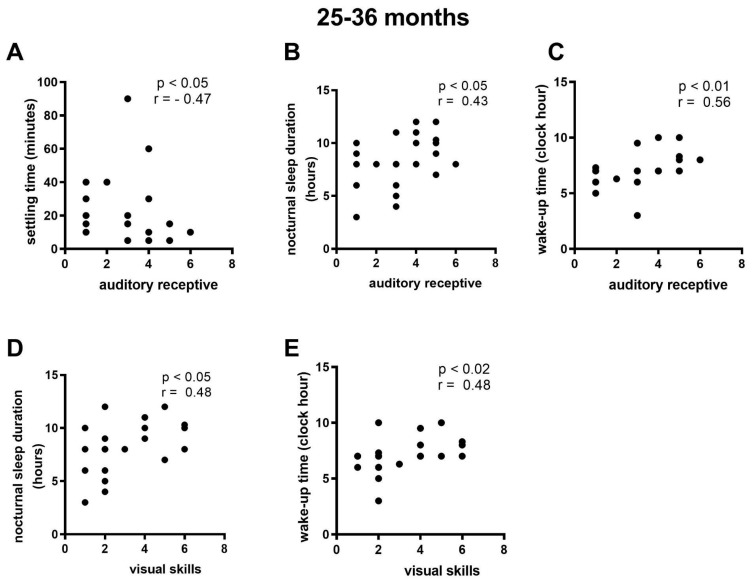
Correlations between the sleep and language parameters in CZS infants with ages ranging from 25 to 36 months. (**A**) Correlation between settling time and auditory receptive skills. (**B**) Correlation between nocturnal sleep duration and auditory receptive skills. (**C**) Correlation Between wake-up time and auditory receptive skills. (**D**) Correlation between nocturnal sleep duration and visual skills. (**E**) Correlation between wake-up time and visual skills. N = 23.

Among children aged 25 to 36 months, the correlation analysis revealed a negative relationship between receptive auditory ability and sleep latency and a positive association with hours of nighttime sleep (Figure 5A,B); a positive relationship between receptive auditory ability and wake time (Figure 5C); and a positive relationship between visual ability and nighttime sleep duration and wake time (Figure 5D,E).

From 37 to 48 months of age, children exhibited the following sleep characteristics: bedtime at 21:30 h [20:25–22:00 h], nighttime wake time 27.5 min [5–110 min]; nighttime sleep duration of 8.5 h [7–9 h]; nighttime sleep latency was 35 min [15–105 min] (Table 4). Among children aged 37 to 48 months, we found no statistically significant correlations between sleep parameters and language development.

Regarding global development, when comparing the groups across different age ranges for the selected skills, there was a decrease in cognitive ability between 13–18 months and 19–24 months (7 [6–10] vs. 5 [4–6], *p* = 0.03) and an increase between 19–24 months and 25–30 months (5 [4–6] vs. 9 [5–10], *p* = 0.01). No child scored in gross motor skills. In fine motor skills, there was an increase between 19–24 months and 25–30 months (2 [1–3] vs. 3 [3–4], *p* = 0.025). In social ability, the subscale related to adaptive behavior, there was a decrease between 25–30 months and 31–36 months (13 [13–15] vs. 12 [9–13], *p* = 0.01).

Correlations were found between expressive language and cognition (*p* = 0.007, r = 0.52) and between expressive language and socioemotional condition (*p* = 0.014, r = 0.48). Regarding receptive language, correlations were also found with cognition (*p* = 0.001, r = 0.63) and socioemotional aspects (*p* = 0.011, r = 0.50). As for the aspects of sleep assessed by the BISQ and global development, correlations were found between the time the child goes to bed and expressive language (*p* = 0.010, r = 0.51), receptive language (*p* = 0.048, r = 0.40), cognition (*p* = 0.032, r = 0.43), and fine motor skills (*p* = 0.005, r = 0.54).

## 4. Discussion

The lack of improvement in the sleep parameters runs counter to what would typically be expected in the normal development of the circadian timing system, suggesting a lack of maturation in this system. This lack of maturation is paralleled by the absence of progress in the overall development of children.

The difficulties in establishing sleep patterns in this population may be due to the severe brain impairment that accompanies this condition, leading to structural deficits and disruptions in connections between important areas involved in sleep control [22,23]. Additionally, motor impairment, as well as potential hormonal, molecular, and immunological alterations, could also play a role in sleep [24,25].

Regarding the association between sleep and language development issues, it is known that the nighttime bedtime routine is a key factor in promoting not only healthy sleep but also overall development and well-being in early childhood [26,27]. Thus, the persistent language development deficits already demonstrated in this population could be exacerbated by the sleep disturbances in children with CZS, as indicated by the data in this study.

Our hypothesis of a correlation between sleep and language skills was confirmed as early as the first year of age, when bedtime and nighttime awakenings were related to expressive auditory ability with a negative impact. This suggests that a later bedtime and more nighttime awakenings may be associated with reduced performance in expressive auditory ability. Such an association has been suggested in studies on auditory performance [28] and the development of receptive and expressive vocabulary [29] in other contexts.

As for the association between sleep and auditory skills, it is known that sleep-related breathing disorders and insomnia can result in impairments in auditory behavior, negatively affecting language development in children, making it difficult to understand and express language [28].

At the age of two, there was a negative relationship between nighttime awakenings and expressive auditory ability, indicating that a higher number of nighttime awakenings may be associated with lower expressive auditory performance.

At three years of age, there was a negative relationship between receptive auditory ability and two sleep factors: sleep latency and nighttime sleep duration. This suggests that longer sleep latency and fewer hours of nighttime sleep may be associated with lower receptive auditory performance. There was also a positive relationship between receptive auditory ability and wake time, indicating that waking up earlier may be related to better performance in this skill.

Visual ability exhibited a positive relationship with both nighttime sleep duration and wake time. This suggests that longer nighttime sleep and waking up later may be associated with better performance in terms of visual ability in these children.

These results also highlight the important role of quality sleep in expressive language development [29] and can contribute to a better understanding of the etiology of language deficits in neurodevelopmental disorders, such as the one studied.

We cannot assert that the lack of establishment of sleep patterns is the most determinative factor in this case, but it would play an important role in language development. Children who have a regular sleep schedule and healthy sleep routines may be more receptive to language learning with improved attention and behavior [30].

During sleep, the brain processes and stores information acquired during the day. This is essential for the development of language skills because the brain organizes and retains the words, sounds, and linguistic structures learned. Children who have poor or insufficient sleep may face difficulties in language consolidation, which can negatively affect their linguistic progress [11,31].

Furthermore, it is essential to recognize that sleep not only affects language development but also plays a fundamental role in overall cognitive development [32]. Children who sleep properly have better attention, memory, and information processing capabilities, as well as auditory, visual, and motor perceptual skills [33,34].

The complex interconnection between sleep, language, and cognitive development described in this study highlights that the etiological basis of CZS imposes upon children with this diagnosis a much larger and more complex phenotypic spectrum than previously described.

## 5. Conclusions

In conclusion, the present study revealed that brain alterations caused by Zika virus infection affect both sleep disturbances and delays in language development. It is possible that sleep disturbance may be a mediating factor in the pathway between CZS and delayed language development, as the three analyzed language skills showed a correlation with sleep parameters.

## Figures and Tables

**Table 1:** General characteristics of children with CZS included in this study (N = 135).

Sex	N	Age (Months)	Gestation Age (Weeks)	Weight (g)	Length	Head Circumference at Birth (cm)
Female	59	23.0 ± 1.1	38.2 ± 0.3	2680 ± 94.3	44.9 ± 0.643	29.8 ± 0.4
Male	76	26.3 ± 1.1	38.8 ± 0.3	2753 ± 96.6	45.6 ± 0.510	29.6 ± 0.4

**Table 2:** Total sleep time (including naps) by age range and percentage of children with CZS below the amount recommended by the American Academy of Sleep Medicine, 2016.

Age Range (Months)	N of Subjects (Female/Male)	Recommended Sleep Time (h)	Median [Interquartile Range] of Sleep Time (h)	% of Children with CZS below Recommended
0–12	10/12	12–16	9.5 [7.5–10]	86.4
13–24	14/19	11–14	10 [8.5–10]	78.8
25–36	23/37	10–13	8 [6–10]	71.7
37–48	12/8	9–12	8.5 [7–9]	80

**Table 3:** Minimum, maximum, median, and interquartile range values for sleep parameters in the 0–12 month and 13–24 month age ranges.

Parameters	0–12 Months (N = 22)					13–24 Months (N = 33)				
	Min	25%	Mean	75%	Max	Min	25%	Mean	75%	Max
Start of the child’s bedtime routine	20.2	21.1	21.4	22.0	25.0	18.0	21.0	21.3	22.3	26.0
Latency (min)	0.2	5.0	10.0	20.0	60.0	5.0	10.0	15.0	30.0	90.0
Bedtime (h)	20.3	21.3	22.0	22.09	26.0	19.0	21.1	22.0	22.7	27.3
Sleeping hours at night (h)	2.0	7.5	9.5	10.0	12.0	5.0	8.5	10.0	10.0	12.0
Time awake at night (min)	0.0	0.0	7.5	37.5	480.0	0.0	0.0	5.0	10.0	60.0
Wake-up time in the morning (h)	0.3	5.5	7.0	8.0	11.0	4.1	6.2	7.3	9.0	11.0
Sleeping hours at day (min)	0.0	15.0	60.0	210.0	480.0	0.0	17.5	120.0	180.0	360.0
Night-time awakenings	0.0	0.0	1.0	2.0	4.0	0.0	0.0	0.0	1.0	3.0

Legend: 26 h means 24 h + 2 h, which means 2 a.m. the next day.

**Table 4:** Minimum, maximum, median, and interquartile range values for sleep parameters in the 25–36 month and 37–48 month age ranges.

Parameters	25–36 Months (N = 60)					37–48 Months (N = 20)				
	Min	25%	Mean	75%	Max	Min	25%	Mean	75%	Max
Start of the child’s bedtime routine	10.0	21.0	21.0	22.0	24.0	20.0	20.2	21.3	22.0	23.0
Latency (min)	0.0	10.0	17.5	40.0	450.0	5.0	15.0	35.0	105.0	300.0
Bedtime (h)	0.0	21.0	22.0	23.0	29.3	20.0	21.4	22.3	23.3	25.0
Sleeping hours at night (h)	0.0	6.0	8.0	10.0	12.0	3.0	7.0	8.5	9.0	11.0
Time awake at night (min)	0.0	0.0	5.0	60.0	450.0	0.0	5.0	27.5	110.0	360.0
Wake-up time in the morning (h)	3.0	6.0	7.0	7.3	11.0	3.0	6.0	6.3	7.7	9.3
Sleeping hours at day (min)	0.0	20.0	105.0	175.0	660.0	0.0	0.0	17.5	90.0	360.0
Night-time awakenings	0.0	0.0	1.0	2.0	5.0	0.0	0.0	1.5	3.0	5.0

## Data Availability

The data that support the findings of this study are available from the corresponding author upon reasonable request.
